# The Investigation of Intermediate Stage of Template Etching with Metal Droplets by Wetting Angle Analysis on (001) GaAs Surface

**DOI:** 10.1007/s11671-010-9790-z

**Published:** 2010-09-28

**Authors:** AA Lyamkina, DV Dmitriev, Yu G Galitsyn, VG Kesler, SP Moshchenko, AI Toropov

**Affiliations:** 1 Rzhanov Institute of Semiconductor Physics SB RAS, Acad. Lavrent'eva Ave. 13, 630090 Novosibirsk, Russia; 2 Novosibirsk State University, Pirogova 2, 630090 Novosibirsk, Russia

**Keywords:** Droplet epitaxy, Local droplet etching, Quantum dots, Atomic force microscopy, Molecular beam epitaxy

## Abstract

In this work, we study metal droplets on a semiconductor surface that are the initial stage for both droplet epitaxy and local droplet etching. The distributions of droplet geometrical parameters such as height, radius and volume help to understand the droplet formation that strongly influences subsequent nanohole etching. To investigate the etching and intermixing processes, we offer a new method of wetting angle analysis. The aspect ratio that is defined as the ratio of the height to radius was used as an estimation of wetting angle which depends on the droplet material. The investigation of the wetting angle and the estimation of indium content revealed significant materials intermixing during the deposition time. AFM measurements reveal the presence of two droplet groups that is in agreement with nanohole investigations. To explain this observation, we consider arsenic evaporation and consequent change in the initial substrate. On the basis of our analysis, we suggest the model of droplet evolution and the formation of two droplet groups.

## Introduction

Droplet epitaxy that has been first implemented by Koguchi et al. [1] to produce quantum dots (QDs) became very popular growth technique in the last years. Due to a wide set of growth parameters besides QDs, it allows to fabricate various nanostructures from nanorings and double rings to quantum dot molecules [2-7]. Recently, a modification of droplet epitaxy was offered [8] to pattern the substrate by etching with metal droplets. Local droplet etching (LDE) is demonstrated to be an efficient tool to form templates for QD fabrication, and it allows one to control their properties in the wide range [9-15]. Also, droplet epitaxy is a promising line of research for producing low-density QDs for single-photon emitter [17,18].

In this paper, we focus on the investigation of the initial stage of droplet epitaxy and study the distribution and the properties of nucleated metal droplets, which are the centers of subsequent QDs formation after arsenic exposure. As droplet and hole properties are determined by formation, ripening and etching processes, their investigation is useful for the understanding of the mechanisms of metal redistribution on the surface.

## Experimental Details

The droplets were grown by molecular beam epitaxy on semi-insulating (001)-oriented GaAs substrates using a Riber32P system with valve arsenic source. The growth parameters were chosen that QDs with long-wave luminescence spectra were obtained before. Since the phase of metal droplets primarily is a part of QDs growth, we used the parameters with which in our previous experiments QDs with long-wave spectra were reproducibly obtained. Two MLs of indium were applied to the GaAs surface without As_4_ flux, and then the sample was immediately quenched. The growth was conducted at temperature of 500°C. To study the possible influence of the deposition rate on droplets' properties, two samples with indium deposition rate of 0.04 and 0.16 Ml/s were grown. A sample with gallium droplets on GaAs was produced also to investigate the materials dependence. The deposition rate of gallium was 0.1 Ml/s.

**Figure 1:**
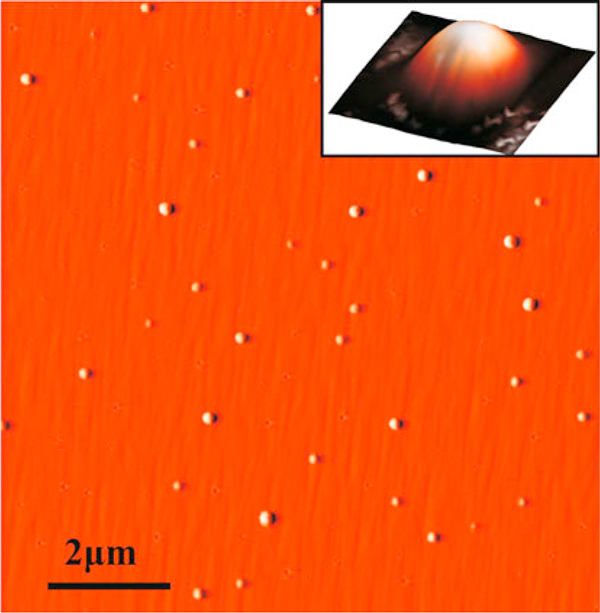
**10 × 10 μm AFM image of GaAs substrate with indium droplets**. The single droplet image 300 × 300 nm is presented in the *inset*.

The morphology of the surface was studied exsitu by the atomic force microscopy (AFM), using a Solver-P47H scanning probe microscope (NT-MDT). Standard silicon cantilevers were used for imaging by AFM. A typical AFM image of indium droplet array is shown in Figure 1.

## Results and Discussion

**Figure 2:**
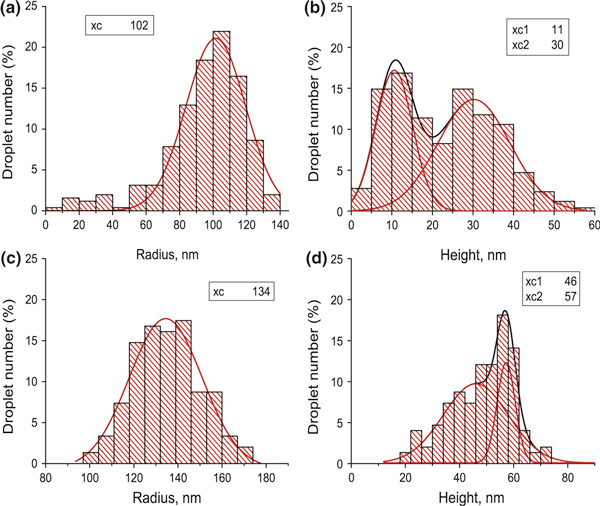
**The histograms of indium droplet geometrical parameter distributions**. The histograms **a** and **b** relate to the sample fabricated with the deposition rate F_In_ = 0.04 Ml/s. The histograms **c** and **d** stand for F_In_ = 0.16 Ml/s. The decompositions into Gaussians are shown, and the values of the *peak centers* are presented in the *insets*.

To study the properties of the droplets, the histograms of the major parameter distributions such as droplet height and radius were plotted. Figure 2 shows the experimental data for the samples with indium droplets.

As it is seen, the distribution of droplet radii is single, while the distribution of their heights has two well-pronounced peaks. This interesting result means that there are two different types of droplets on the substrate that may indicate that the droplet nucleation is a double-stage process, and during the indium deposition time, the initial surface changes significantly.

The comparison of the height distributions at different deposition rates and consequently deposition times in Figure 2 evidences that material redistribution on the surface goes actively during this period. With growth rate increased by a factor of four, the height distribution stays bimodal, but the peaks qualitatively change. Therefore, the growth rate does influence the mechanism of the droplet formation.

To investigate the form of the droplets, we used the distributions of the aspect ratio, denoted further as *γ*, which is the ratio of the droplet height to its radius. Since the lateral droplet size is much larger than the height, the aspect ratio is assumed to be an estimation of the droplet wetting angle. At a high temperature of 500°C, a GaAs substrate can dissolve in the droplet interface region (etching process), and due to the diffusive fluxes, there should be material exchange at the interface mostly caused by the diffusion of liquid gallium to the metal droplet (intermixing process). Due to these processes, the composition of the droplets should therefore differ from that of the original material, and consequently the wetting angle of the droplet changes. The investigation of the wetting angle behavior gives us an additional opportunity to analyze and verify the complex processes of the etching.

**Figure 3:**
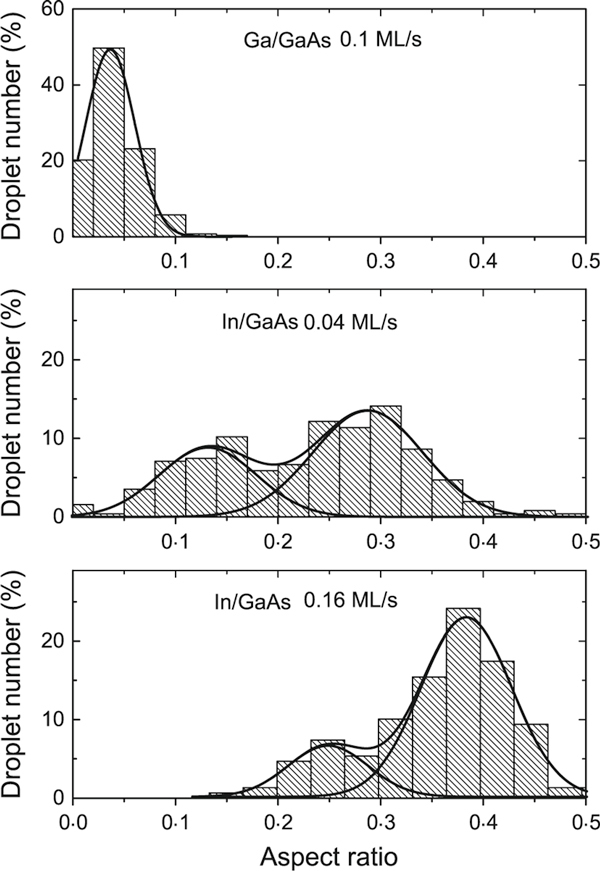
**From *up* to *bottom***: aspect ratio distributions for the samples with gallium droplets and indium droplets fabricated with F_In_ = 0.04 and 0.16 Ml/s, correspondingly. The decompositions into Gaussians are shown.

The histograms of the aspect ratio for the samples with both indium and gallium droplets are presented in Figure 3. As it is seen, the distributions related to the indium droplets are bimodal and in comparison with the height and radius distributions, they demonstrate even more pronounced bimodal character. Therefore, we can suppose that the droplet geometry is the same within each group, but quite different in the two obtained groups.

Considering the etching rate to be almost constant, one can assume that the indium content is higher in the sample with the higher deposition rate. Figure 3 evidences that as the growth rate is increased, the peak shifts to larger wetting angles and that the peaks of all indium droplets correspond to larger aspect ratios than the only peak of gallium droplets. We therefore conclude that during the deposition time, gallium that has dissolved from the substrate mixes with indium metal droplet and the composition changes from pure indium to In_*x*_Ga_1-*x*_. Since the aspect ratio measured experimentally correlates with the wetting angle, we can estimate the degree of the intermixing with the indium content *x*.

The wetting angle is determined through the surface tension by the Young equation [19]:

$$\cos\theta =\frac{\sigma_{\text{sg}}-\sigma_{\text{sl}}}{\sigma_{\text{gl}}}$$

where *θ* is the contact angle, σ_sg_, σ_sl_ and σ_gl_ are the surface tensions on the solid–gas, solid–liquid and gas–liquid boundaries, correspondingly. As the droplet composition influences σ_sl_ much stronger than σ_gl,_ we neglect the contribution of the gas–liquid boundary tension and consider the wetting angle to be proportional to σ_sl_.

For numerical estimation of the indium content *x* in In_*x*_Ga_1-*x*_ droplets, we suppose a linear connection between *x* and *γ*: *x* = *a γ* + *b*. To determine the constants *a* and *b*, we assumed *x* = 0 for gallium droplets with γ = 0.04 and *x* ~ 1 for the largest indium content in our experiments (the deposition rate of 0.16 Ml/s, right peak in Figure 3); the values of *a* and *b* were found to be 2.94 and -0.12, respectively. The results for the intermediate values of *γ* are presented in Table 1.

**Table 1:** 

γ	0.38	0.29	0.25	0.13	0.04
*x*	1	0.74	0.62	0.27	0

We therefore can conclude that material intermixing that occurs during the deposition time is very significant.

Using these results, we then can estimate the depth of the nanoholes etched by indium in the framework of the following scheme. According to our assumptions, the droplets with *γ* = 0.38 consist of pure indium. The droplets with different γ contain some gallium that comes from the dissolved substrate and intermixes with indium. These droplets are supposed to originate from pure indium droplets with the sizes measured by AFM, so the amount of indium was determined. With the experimentally observed wetting angle and estimated indium content, we can calculate the amount of gallium in the droplet and the corresponding volume of dissolved GaAs to provide it. Then, assuming some profile of the substrate etching, it is possible to determine the depth of the formed nanoholes.

**Figure 4:**
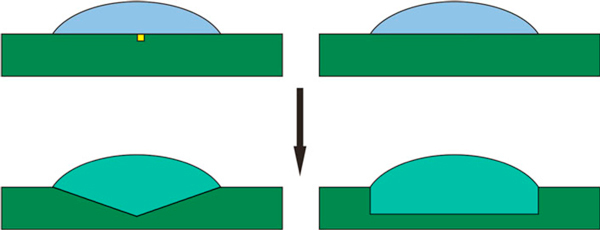
**The sketch of the evolution of metal droplet on the stable substrate (*right*) and in presence of a defect (*left*), resulting in *rectangular* and *triangular* profiles, respectively**.

We made the estimations for two simple models that are based on the nanohole investigation in the reference [15], where two groups of the nanoholes were observed that were called deep and flat due to the depth difference. Simplifying the nanohole geometry, we assume the etching profile to be rectangular or triangular (Figure 4). The first model might correspond to a low etching rate in the absence of any surface defect and consequently to a stable substrate providing uniform etching under the droplet. The second model describes the case of defect etching with a high rate, so the etching volume is limited by the crystal plates.

The droplet with a height of 50 nm and a radius of 200 nm (volume of ~1.5 × 10^6^ nm^3^) was considered to be a typical droplet consisting of pure indium as it was mentioned above. For γ = 0.25 (*x* ~ 0.6), GaAs volume of 1.8 × 10^6^ nm^3^ was found that results in the nanohole depth of 15 and 45 nm for cylindrical and conical holes, respectively. The latter value is close to the one obtained for nanoholes in the reference [16] for a high indium content in the etchant. We can suppose that under such droplets, there are conical etched volumes corresponding to the deep nanoholes. Such a simplified consideration can be useful for a further detailed investigation of the connection between the droplets and the nanoholes.

**Figure 5:**
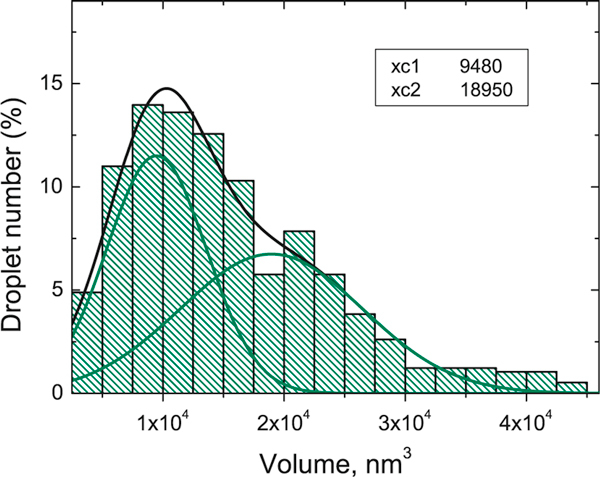
**The histogram of a volume distribution for gallium droplets**. The decomposition into Gaussians is shown, and the values of the *peak centers* are presented in the *inset*.

While gallium droplets certainly demonstrate a single peak of the aspect ratio, their volume distribution reveals the coexistence of large and small droplets varying by the volume approximately by a factor of two, as it is seen in Figure 5. The presence of the two pronounced groups of droplets should lead to the subsequent formation of the bimodal nanohole distribution that was obtained in some experiments [12,15]. Estimating the volume ratio of the deep and flat holes fabricated by the etching with gallium droplets in [15], we found that it is in a good agreement with the droplet volume ratio in our experiment.

**Figure 6:**
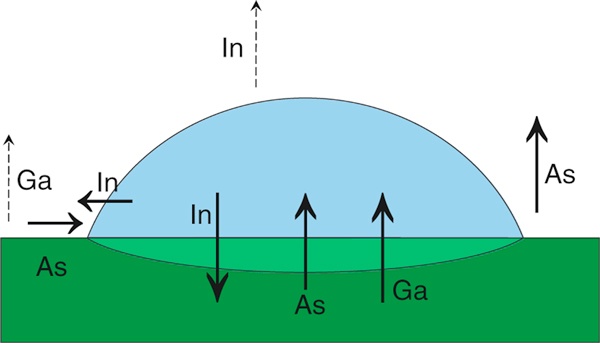
**The scheme of indium droplet on GaAs substrate considered as a three-phase system**. The material fluxes are marked with the *arrows*. The *dashed arrows* refer to the fluxes of gallium and indium.

The bimodality effect was obtained before for different parameters of the nanoholes [12,15,16] that are the final result of local droplet etching. Having revealed the bimodality of initial droplet parameters, we suppose that all bimodalities are a consequence of some fundamental mechanisms of droplet formation and evolution in the early stage of LDE. To analyze these processes within thermodynamics approach, we consider the droplet, the substrate and chamber volume as a three-phase system according to the scheme presented on Figure 6. This general scheme can be used for both the process of growth where we take into account simultaneous etching and the post-growth period when an external indium flux should be excluded.

The diffusion flows on the interface between the liquid and solid phases are determined by etching and intermixing processes, and they have a key role in the droplet evolution. The equation of material balance for the droplet has a form:

(1) $$V+V^{\text{WL}}=F^{\text{In}}t+F_{\text{diff}}^{\text{Ga}}t-F_{\text{diff}}^{\text{In}}t-\Delta V_{\text{Ga}}t$$

where *F*^In^ stands for an external flux, $F_{\text{diff}}^{\text{In}}$, $F_{\text{diff}}^{\text{Ga}}$ are the diffusive fluxes, *V* is the droplet volume, *V*^WL^ is the volume of the metal wetting layer, Δ*V*_Ga_ is the rate of etching related to the specific change of gallium volume that takes place when solid GaAs dissolves to the liquid metal and arsenic under the droplet. The external indium flux is fixed with the growth conditions, while the others can be time dependent due to the changes in the system. *F*^In^*t* is the total amount of indium supplied to the substrate that was the same for both samples with indium droplets.

Our measurements revealed that the droplet volumes per square micron are *V*_1_ ~ 4.3 × 10^5^ nm^3^ for the deposition time of 12 s and *V*_2_ ~ 1.5 × 10^5^ nm^3^ for 50 s. The total amount of the deposited indium is *V* = 12.7 × 10^5^ nm^3^, so it is seen that *V*_1_ and *V*_2_ differ from *V* significantly, and we can expect that a part of material is stored in the wetting layer. The formation of the wetting layer is confirmed by photoluminescence measurements for QDs fabricated by droplet epitaxy in the same growth conditions [20]. A clear peak corresponding to the quantum well and consequently wetting layer was obtained in the spectra measured at 4°K. For quantitative analysis, Auger measurements were conducted. Taking into account the AFM data on the QD sizes, we used the model of infinitely thick QDs (the height is much larger than the electron inelastic mean free path) on the surface with a thin wetting layer. The thickness was assumed to be ~1 nm as about the critical value in the Stranski–Krastanov growth mode. The area of the wetting layer was found to be 90% of the total surface, so it is almost uniform and may contain a significant part of deposited material playing a role in (1).

Concerning droplet nucleation, the fluxes on the boundary with vacuum chamber (solid–gas and liquid–gas interfaces) should be considered. There is an uncompensated arsenic flux from the substrate to the chamber, so arsenic evaporation can result in the appearance of additional nucleation centers. It is interesting to notice that the droplet densities were 4.3 × 10^7^ and 3.75 × 10^7^ cm^-2^ for the deposition rates of 0.04 and 0.16 Ml/s, respectively, so the increase of the deposition rate and time from 12 to 50 s does not change the droplet density considerably.

Besides our experimental data, the results of the investigation of the nanoholes fabricated by LDE can be used for the analysis. In the reference [15], the nanohole groups with different geometries were obtained and the possibility of the nanohole formation through different mechanisms was supposed to explain their presence. Taking into account the connection between the initial droplets and the holes formed by the etching, there should be two mechanisms of the droplet formation. The difference obtained for the droplet volumes allows us to assume that the droplets form at different time instants. The appearance of additional nucleation centers leading to the nucleation of the small droplets can be caused by arsenic evaporation (uncompensated arsenic flux in Figure 6 that is usually neglected in the LDE approach) and a consequent occurrence of surface defects. In our experiment, the deposition time is quite long in comparison with that usual for LDE, so this effect might be more pronounced. The appearance of the defects can be monotonic in time or critical because of a possible surface reconstruction phase transition of the order–disorder type caused by the arsenic evaporation [21].

In the first case, only one droplet group is expected and the droplet density should be proportional to time, which contradicts our data. In the case of a reconstruction transition provided by an arsenic surface content change, a jump of the droplet density is expected, which is not obtained for the deposition times of 12 and 50 s.

The presence of two droplet groups can be provided by the existence of two stable compounds of the etchant like In_*x*_Ga_1-*x*_. The bimodality of the aspect ratio is obtained experimentally, but for gallium droplets with a single wetting angle, there are two groups of droplets varying in the volume. The existence of two droplet groups can be explained with the presence of two kinds of surface defects that are etched in different ways, so that the droplets can differ in the composition, volume and geometry. The origin of these defects should be found out.

Considering our experimental data on the droplet properties and the published results of etched nanohole investigations, we suppose the following scheme of the formation of a metal droplet:

1. The flux of a metal is applied to the substrate and the droplets form on the initial nucleation centers present on the surface.

2. During the deposition time, arsenic evaporates and the substrate surface state changes. A surface reconstruction transition occurs, and additional nucleation centers arise.

3. The droplets evolve; due to different nucleus origin, their etching proceeds differently. For indium as an etchant, we expect the presence of metal droplets with two different indium contents. This is corroborated by the bimodality of the aspect ratio obtained in the experiment. For gallium as an etchant, there should be two groups of droplets formed at different moments of time and etched with different rates. Experimentally, we observed two droplet groups differing in the volume.

## Conclusions

In this work, the array of metal droplets on a semiconductor surface is studied as an initial stage of LDE and droplet epitaxy. The samples with various droplet materials and deposition rates were grown. We have observed a bimodality of the droplet height distribution in the system of indium droplets on a GaAs substrate that is similar to the hole depth bimodality shown in the reference [15]. It indicates that the droplet formation proceeds in two stages.

A new method to investigate the intermixing process by a wetting angle analysis is proposed. We used the aspect ratio which is defined as the ratio of the droplet height to its radius as an estimation of the wetting angle depending on the materials. A bimodality of the aspect ratio was found, which indicates the existence of two droplet groups with different compositions. The investigation of the wetting angle and the estimation of indium content revealed a significant material intermixing during the deposition time.

Based on our experimental results and the nanohole investigations in the references [15,16], the existence of two droplet formation mechanisms is suggested. Taking into account the arsenic evaporation and a consequent change of the substrate surface state, we suggested the model of droplet evolution and the formation of two droplet groups.
